# Neuroprotection Targeting Protein Misfolding on Chronic Cerebral Hypoperfusion in the Context of Metabolic Syndrome

**DOI:** 10.3389/fnins.2018.00339

**Published:** 2018-05-31

**Authors:** María I. Herrera, Lucas D. Udovin, Nicolás Toro-Urrego, Carlos F. Kusnier, Juan P. Luaces, Matilde Otero-Losada, Francisco Capani

**Affiliations:** ^1^Centro de Investigaciones en Psicología y Psicopedagogía, Facultad de Psicología y Psicopedagogía, Universidad Católica Argentina, Buenos Aires, Argentina; ^2^Instituto de Investigaciones Cardiológicas (ININCA), Universidad de Buenos Aires (UBA-CONICET), Buenos Aires, Argentina; ^3^Facultad de Medicina, Universidad Católica Argentina, Buenos Aires, Argentina; ^4^Universidad Autónoma de Chile, Santiago de Chile, Chile

**Keywords:** metabolic syndrome, chronic cerebral hypoperfusion, neuroprotection, protein misfolding, endoplasmic reticulum stress, chaperones, neurodegenerative diseases

## Abstract

Metabolic syndrome (MetS) is a cluster of risk factors that lead to microvascular dysfunction and chronic cerebral hypoperfusion (CCH). Long-standing reduction in oxygen and energy supply leads to brain hypoxia and protein misfolding, thereby linking CCH to Alzheimer's disease. Protein misfolding results in neurodegeneration as revealed by studying different experimental models of CCH. Regulating proteostasis network through pathways like the unfolded protein response (UPR), the ubiquitin-proteasome system (UPS), chaperone-mediated autophagy (CMA), and macroautophagy emerges as a novel target for neuroprotection. Lipoxin A4 methyl ester, baclofen, URB597, N-stearoyl-L-tyrosine, and melatonin may pose potential neuroprotective agents for rebalancing the proteostasis network under CCH. Autophagy is one of the most studied pathways of proteostatic cell response against the decrease in blood supply to the brain though the role of the UPR-specific chaperones and the UPS system in CCH deserves further research. Pharmacotherapy targeting misfolded proteins at different stages in the proteostatic pathway might be promising in treating cognitive impairment following CCH.

## Introduction

The energy requirements of the brain are high, and neuronal viability critically depends on cerebral blood flow (CBF) delivery of oxygen and nutrients (Daulatzai, 2017). Endothelial cells interact with pericytes, glial cells, and neurons to coordinate functions in a neurovascular unit (NVU) (Hermann and ElAli, 2012). Endothelial dysfunction is typically associated with metabolic syndrome (MetS) (Otero-Losada et al., 2013, 2014), and affects CBF distribution and NVU integrity (McCrimmon et al., 2012) bringing about chronic cerebral hypoperfusion (CCH). CBF impairment increases the risk of cognitive decline shaping a neurovascular pathway to sporadic Alzheimer's disease (AD) (Zlokovic, 2011). Since an adequate protein folding and trafficking in neurons depends on energy supply by CBF (Wang and Kaufman, 2016), protein misfolding might be considered a mechanism linking CCH with AD. On these grounds, targeting proteome homeostasis portrays a promising neuroprotective approach in AD prevention (Jackrel and Shorter, 2017; Sweeney et al., 2017) in the context of MetS.

## Chronic cerebral hypoperfusion as a silent consequence of MetS

MetS is the constellation of vascular risk factors including hypertriglyceridemia, hyperglycemia, and/or insulin resistance, hypertension, and visceral obesity in man (Otero-Losada et al., 2016). These factors embody the prelude to type 2 diabetes (T2D) (Bruce and Hanson, 2010), characterized by hyperglycemia, hyperinsulinemia and low insulin sensitivity (American Diabetes Association, 2002; McCrimmon et al., 2012). Type 2 diabetes is usually concurrent with several features of the MetS, which contribute to its severity (McCrimmon et al., 2012). Defective angiogenesis in T2D leads to immature vascularization (Li et al., 2010), CCH, neuro-glial dysfunction, and degeneration in time (ElAli et al., 2013). Endothelial injury and microvascular dysfunction associated with MetS (McCrimmon et al., 2012) narrows luminal spaces during sustained periods of high metabolic demand (de la Monte, 2014) leading to CCH and cerebral metabolic suffering (Daulatzai, 2017; Obadia et al., 2017).

Insufficient brain perfusion in CCH is chronic, silent, and may last for years, progressively damaging brain tissue and should be distinguished from sudden CBF obstruction due to brain ischemia (de La Torre, 2008; de la Torre, 2012). Besides, moderate hypoperfusion (a 30% CBF rate decrease in De Jong et al., 1999) and mild neuronal damage observed in experimental models of CCH (Farkas et al., 2007) contrast with the full CBF deprivation found in acute ischemia models (Jia et al., 2015; Park and Lee, 2017).

Clinical studies on the circulatory impact of MetS factors' prevalence show that progressive clustering of MetS factors escalate microvascular damage due to further weakening of the cerebral arterial vasodilatation response (Nazzaro et al., 2013). The concurrence of three or more MetS features leads to a substantial CBF decrease mainly in the mediolateral areas of the frontal, parietal, temporal, and occipital gray matter and weakening of the immediate memory (Birdsill et al., 2013), attention, processing speed, executive functions, fluid intelligence, and visuospatial processing (Dik et al., 2007; Muller et al., 2010; Reijmer et al., 2011). Accordingly, on cerebral perfusion matters, MetS should be studied as a whole rather than as the mere concurrency of multiple scattered factors (Mellendijk et al., 2015).

A systematic revision of longitudinal population-based studies on the contribution of different MetS features to the risk of dementia confirmed an association as for hypertension, dyslipidemia, obesity, and T2D, the two latter imposing the greatest risk (Kloppenborg et al., 2008). The progressive brain damage associated with T2D is known as diabetic encephalophathy (Van den Berg et al., 2007). However, identifying the actual factors responsible for diabetic encephalophathy is hampered not only by the presence of multiple vascular comorbidity factors in MetS (McCrimmon et al., 2012) but also by co-variables like glycemic control, disease history, and treatment modality in T2D (Van den Berg et al., 2007).

Murine models of MetS and T2D showed time-dependent cerebrovascular impairment. Mice fed a high-fat diet (HFD) for 12 weeks developed arteriolar damage in the brain and CBF alteration (Lynch et al., 2013), and cerebral endothelial dysfunction appeared around the week 5 in a mouse model of streptozotocin-induced diabetes (Kitayama et al., 2006). In both studies, the endothelial dysfunction in cerebral arterioles preceded that in the carotid arteries (Kitayama et al., 2006; Lynch et al., 2013). We reported carotid damage in another mice model 8 weeks after MetS induction (Otero-Losada et al., 2013), so time-dependency may vary upon the experimental settings. Recent evidence revealed that aging exacerbated cerebrovascular alterations in the hippocampus and the cerebral cortex that ushered cognitive impairment in HFD mice (Tucsek et al., 2014). Furthermore, cells exposed to hypoxia and high glucose underwent autophagy dysregulation, and impaired mitochondrial quality control, reproducing CCH and diabetes *in vitro* (Song et al., 2018).

## Chronic cerebral hypoperfusion and sporadic ad

Long before cognitive decline becomes apparent, CCH stands for an early sign of sporadic AD (Daulatzai, 2017; de la Torre, 2017). In this regard, the sporadic AD has been described as a vasocognopathy, a vascular-related cognitive disorder (de La Torre, 2004) upon CCH pathogenic requirement (Austin et al., 2011). The long-standing decline in cerebral circulation triggers a neuronal energy crisis and a pathogenic cascade giving way to the characteristic cognitive decline (de La Torre, 2008) in CCH (Tanashyan et al., 2016).

As sporadic AD shows aggravated hypoperfusion from the pre-clinical phases to the advanced stages with the progression of the disease, CCH may represent a promising biomarker in the early diagnosis of AD (Austin et al., 2011). In this regard, the interest on the critical role of vascular risk factors like hypertension, hypercholesterolemia, and diabetes, and the ensuing CCH (Austin et al., 2011) in the early stages of the sporadic AD (Chen et al., 2011) has lately increased. The primary CBF deficiency concept has replaced that of secondary deficiency aiming to better understand the initial memory loss in AD (Mazza et al., 2011).

Most studies have focused on oxidative stress and neuroinflammation to explain the association between CCH and AD (Zhao and Gong, 2015). Beyond them, protein misfolding and aggregation emerges as a novel relevant mechanism (Jackrel and Shorter, 2017). Extraneuronal accumulation of β-amyloid peptide (Aβ) is found in the senile plaques long before cognitive AD deficits. Distinctively, intraneuronal tau protein aggregates in neurofibrillary tangles (NFTs) appear later upon clinical progression impairing axonal transport and synaptic function (Ashraf et al., 2014).

Not only neuronal cells are particularly vulnerable to protein aggregation, but also their unique cellular structure precludes protein quality control. Post-mitotic neurons are unable to remove cytotoxic proteins after cell division (Ciechanover and Kwon, 2015), and protein aggregates in dendrites and axons need to be packaged into autophagic vacuoles to return to the cell body for lysosomal degradation. While aging slows-down protein quality control systems (Ciechanover and Kwon, 2015), age-related stress and protein misfolding play a major role in cerebral proteopathies, the sporadic forms of the neurodegenerative disease (Saxena and Caroni, 2011). MetS is a high-risk condition for premature aging-related changes (Otero-Losada et al., 2011, 2016).

## Proteome homeostasis and neurodegenerative protein disorders

### ER stress and misfolded proteins clearance mechanisms

**Figure 1:**
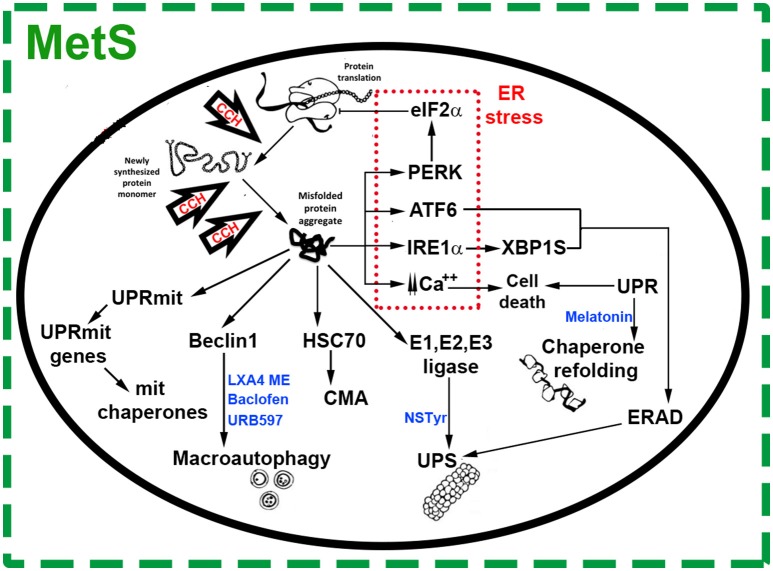
Chronic Cerebral Hypoperfusion (CCH) induces protein misfolding in the context of Metabolic Syndrome (MetS). Under cell stress, proteostasis network surveillance systems refold or degrade proteins through several mechanisms. Accumulation of misfolded proteins in the Endoplasmic Reticulum (ER) triggers the unfolded protein response (UPR) which induces an upregulation in the expression of chaperone genes. This response is characterized by transcription factor 6 (ATF6), inositol-requiring enzyme 1a (IRE1-α) and X box–binding protein 1 (Xbp1). These ER stress components trigger ER-associated degradation (ERAD) genes, which work to restore organelle function and maintain cell survival. Under ER stress, protein RNA-like ER kinase (PERK) mediates the phosphorylation of eukaryotic translation initiator factor 2α (eIF2α), which inhibits translation and attenuates protein synthesis at the ER. Prolonged ER stress increases levels of intracellular calcium, activating signals of cell death. Mitochondria responds to cell stress throughout its own protein quality control system known as UPR(mt), promoting transcription of mitochondrial chaperones and factors for organelle homeostasis. Depending on the nature, size and solubility of substrates, misfolded proteins can also be degraded by the Ubiquitin Proteasome System (UPS), Chaperone Mediated Autophagy (CMA) or macroautophagy. In general, most soluble and monomeric misfolded proteins are degraded by the UPS and CMA. Substrates targeted by CMA are bound by chaperone heat-shock cognate 70 (Hsc70) and degraded into amino acids by lysosomal hydrolases. Misfolded proteins in the cytosol are also conjugated to ubiquitin through an enzymatic cascade involving the ubiquitin-activating (E1), ubiquitin-conjugating (E2), and ubiquitin-protein (E3) enzymes. Then, ubiquitinated substrates are degraded by the proteasome. Other substrates, such as protein aggregates, are recognized by molecular chaperones, ubiquitinated and delivered to the autophagosome via Beclin-1 complex. The autophagosomes are fused with lysosomes to form autolysosomes, where misfolded proteins are degraded. Agents targeting proteostasis network pathways in CCH-induced protein misfolding are shown in blue color text. N-stearoyl- L-tyrosine (NSTyr), Lipoxin A4 methyl ester (LXA4 ME).

The endoplasmic reticulum (ER) plays a pivotal role in the high energy-demanding protein folding and trafficking processes. Energy restriction under stressing conditions leads to unfolded or misfolded proteins' accumulation in the ER lumen (Wang and Kaufman, 2016). In this scenario, triggering the unfolded protein response (UPR), an adaptive function of protein quality control that reduces polypeptide synthesis, improves correct protein folding, and promotes misfolded protein degradation, restores cell homeostasis avoiding apoptosis (Sims-Robinson et al., 2016; Figure 1). Three main signaling pathways are activated under ER stress conditions: the inositol-requiring enzyme 1a (IRE1α), the protein RNA-like endoplasmic reticulum kinase (PERK), and the activating transcription factor 6 (ATF6) (Lindholm et al., 2017; Figure 1). The endoribonuclease IRE1α produces an active form of the transcription factor X-box binding protein-1 (XBP-1) triggering the UPR which upregulates chaperone genes involved in protein folding (Lindholm et al., 2017). Chaperones help new proteins in their timely degradation and adequate folding, without influencing their final structure (Balchin et al., 2016). In this way, proteostasis or proteome functional homeostasis is partially restored (Lindholm et al., 2017; Figure 1). Conjoinctly, ER stress activates PERK which phosphorylates the eukaryotic translation initiation factor-2α (eIF2α), down-regulating protein synthesis and decreasing misfolded proteins in the ER (Figure 1). Finally, under ER stress conditions, ATF6 migrates to the nucleus and activates genes of ER chaperones (Cybulsky, 2013).

Endoplasmic reticulum stress also increases protein degradation in the ER lumen and membrane by way of the ER-associated degradation (ERAD) mechanism which, together with soluble cytoplasmatic misfolded proteins, produce the activation of clearance mechanisms such as the ubiquitin (Ub)-proteasome system (UPS) (Figure 1). In eukaryotic cells, the UPS is the most important degradation pathway for a broad range of short-lived proteins which regulate cellular processes and those of quality control of protein folding and proteotoxic stress. The main protease of the UPS is the proteasome which degrades substrates bearing a poly-ubiquitin chain (Chowdhury and Enenkel, 2015; Ji and Kwon, 2017; Figure 1).

Chaperone-mediated autophagy (CMA), another line of defense against misfolded proteins, is a branch of the autophagy-lysosome system (Figure 1). The heat-shock cognate 70 (Hsc70) chaperone recognizes misfolded proteins exposing the KFERQ degradation signal, luring them to lysosomes for degradation by hydrolases (Ciechanover and Kwon, 2015).

Substrates escaping the surveillance mechanisms are not vulnerable to the mentioned proteolytic pathways and tend to form aggregates, which are mainly removed by macroautophagy (Figure 1). Misfolded protein substrates of macroautophagy are recognized by molecular chaperones, ubiquitinated, and delivered into autophagosomes, which are later incorporated to lysosomes and undergo degradation (Ciechanover and Kwon, 2015).

Misfolded proteins also trigger the UPR (mt) mitochondrial response, characterized by distinctively own protein quality control system (De et al., 2018) whereby nuclear transcription of mitochondrial chaperones takes place for organelle homeostasis (Lindholm et al., 2017; Figure 1).

All the above-mentioned quality control systems, a collection of chaperones and protein degradation machinery working to balance the proteome, embody the proteostasis network (Figure 1). Nevertheless, there are some aggregated proteins resistant to quality control systems representing a common molecular mechanism reported for a group of so-called protein misfolding disorders (PMD) like the AD, Parkinson's disease (PD), Huntington's disease (HD), and others (Ciechanover and Kwon, 2015). A defective ER regulation of many cellular processes may contribute to the progression of the pathology (Lindholm et al., 2017) while proteostasis alteration leading to chronic activation of the UPR and other surveillance mechanisms may contribute to the pathogenesis of many diseases (Ozcan and Tabas, 2012).

The deposition and accumulation of misfolded proteins is a common sign in many neurodegenerative diseases, characterized by cell signaling impairment and defective neuronal connectivity following cell death (Soto, 2003). Under cellular ER stress, clearance and protein refolding pathways are activated while the UPS and other systems are mitigated by protein aggregates or toxic products including reactive oxygen species (Soto, 2003). The dysfunctional UPS caused by the accumulation of proteins in the cell furthermore aggravates ER stress (Ciechanover and Brundin, 2003). Finally, altered proteostasis in this stressful cell environment affects mitochondria, caspases are activated, and cell death ensues (Rao et al., 2004).

### ER stress in Alzheimer's disease and other proteopathies

The hallmarks of Alzheimer's disease (AD) are the aggregation of extracellular amyloid-β peptides and intracellular phosphorylated tau proteins, and the abnormal intracellular calcium levels with neuronal deterioration, that lead to death (Ozcan and Tabas, 2012). The brain of deceased AD patients showed activated UPR expressing chaperone Grp78 (Hoozemans et al., 2005), and immunohistochemical evidence of PERK and eIF2α activation (Unterberger et al., 2006; Scheper and Hoozemans, 2015). Inhibiting PERK decreased p-eIF2α levels and partially reversed memory impairments in an AD mouse model (Ma et al., 2013). The evidence unquestionably confirms the specific ER signaling effect on neurons and neuronal connections not only in the AD but also in other PMD like Parkinson Disease (PD) in which UPR dysfunction has been currently reported. Mutations in the Parkin gene impaired the degradation of unfolded proteins (Ciechanover and Kwon, 2015). Also, the accumulation of a substrate from Parkin gene led to ER stress and apoptosis, and phosphorylated forms of PERK (p-PERK) and eIF2α (p-eIF2α) increased in PD neurons (Ozcan and Tabas, 2012).

## Experimental findings of CCH-induced neurodegeneration by protein misfolding

The impact of CCH on neurodegeneration has been investigated using different animal models (Zhao and Gong, 2015). Increasing evidence shows that protein misfolding is involved in CCH-induced neurodegeneration. Both a bilateral common carotid artery occlusion (BCCAO) murine CCH model (Ozacmak et al., 2009), and another animal model of CCH designed to reproduce human hemodynamic insufficiency (Hai et al., 2002) showed a compensatory defensive neuronal loss in the hippocampal CA1 region. Years later, ultrastructural analysis using ethanolic phosphotungstic acid (EPTA)-stained electron microscopy confirmed that neurodegeneration was induced by protein aggregation after protein synthesis machinery destruction (Jian et al., 2013).

The stress-sensitive and novel negative modulator of myelination Redd1 (RTP801/Dig2/DDIT4) expresses in response to hypoxia, ER stress, and autophagy. It underwent time-dependent changes in a rat CCH model induced by permanent BCCAO and two-vessel occlusion (2VO) (Park and Lee, 2017). Following surgery, Redd1 increased in pyramidal neurons of the CA1 region by day 7 and gradually decreased by day 28, being associated with CCH-induced neuronal damage (Park and Lee, 2017). Autophagy upregulation by CCH in bilateral common carotid artery ligation (BCCAL) and oxygen-glucose deprivation (OGD) models associated with brain atrophy and neuronal apoptosis in the CA1 area (Liu et al., 2015). Likewise, neuronal damage and high level of the autophagy markers LC3-II and beclin-1 were found in the CA1 area after CCH, along with ultrastructural evidence of an increased number of apoptotic neurons showing the typical pyknotic nuclei, autophagosomes and autolysosomes, and of ER fragmentation among other changes. These observations have been correlated with the spatial working memory impairment (Wang et al., 2017).

Cognitive and pathological features and the associated autophagic modifications were evaluated from week 2 to 20 after BCCAO to elucidate the long-term neurological effects of CCH-induced autophagy after CBF recovery, and CBF changes were monitored in a two-step BCCAO rat model. Unlike the sustained increase in the autophagy markers Beclin-1, light chain 3B, and P62, CBF returned to baseline. Regardless CBF recovery, a striking cognitive decline, and neuronal damage were observed revealing the early contribution of the early autophagy impairment to the later neurodegeneration and cognitive decline. Autophagic dysfunction may hamper the successful clearance of the Aβ peptide, leading to cognitive alterations. Other interesting findings suggested that cortical neurodegeneration and autophagic changes precede those in the hippocampus, the same as white matter damage precedes gray matter degeneration. However, only hippocampal deposition of Aβ plaques was related to cognitive failure (Zou et al., 2017). The hippocampus is the archetypical brain area of learning and memory that becomes dysfunctional in AD (Ashraf et al., 2014), and the CA1 area is specifically vulnerable to hypoperfusion (Liu et al., 2015). Hippocampal neurodegeneration also developed in a rat model of BCCAL and 2VO (Jia et al., 2015). Sustained ER stress due to CCH was inferred from the expression of the CCAAT/enhancer binding protein, C/EBP, homologous protein. Besides, CCH stimulated macroautophagy based on the ratio of microtubule-associated protein light chain 3 II (LC3-II) to LC3-I and beclin1 marker level (Jia et al., 2015).

Over and above, dysfunction of the UPS might be related to hippocampal CA1 degeneration after CCH as concluded after finding long-term locally decreased proteasome peptidase activity, and accumulation of ubiquitinated protein aggregates in a rat model (Hai et al., 2013). Earlier studies had reported similar results suggesting that the reduced activity of the UPS might impair the removal of misfolded proteins leading to protein aggregation and eventual neurodegeneration (Hai et al., 2011). The following cognitive impairment might compromise both learning and spatial memory skills concurrently with long-term potentiation (LTP) inhibition (Hai et al., 2009). Right common carotid artery permanent ligation also induced protein aggregation and mild CCH resulted in NVU dysfunction and rapid Aβ deposition in ipsilateral brain capillaries (ElAli et al., 2013). The same as observed in amyloid protein precursor (APP)- transgenic (APP-Tg) mice, bilateral carotid artery stenosis (BCAS) induced CCH-accelerated Aβ deposition. Accordingly, CCH might precipitate the neurodegenerative process in AD (Kitaguchi et al., 2009). Previous findings in a rat model of BCCAL and 2VO demonstrated that CCH fostered the aberrant processing of APP (Bennett et al., 2000). Unilateral common carotid artery occlusion (UCCAO) resulted in CCH and induced tau hyperphosphorylation, memory deficits, dysregulation of synaptic proteins, and decreased post-translational tau O-GlcNAcylation by β-N-acetylglucosamine (Zhao et al., 2014). Earlier studies had suggested that brain glucose metabolic dysfunction down-regulated tau O-GlcNAcylation mediated by tau hyperphosphorylation (Liu et al., 2004; Liu F et al., 2009; Liu Y et al., 2009).

CCH in AD leads to increased tau protein hyperphosphorylation and intracellular aggregation upon conformational changes (Šimić et al., 2016; Lathuilière et al., 2017), same as found after UCCAO in mice or 2VO in rats (Li et al., 2015; Qiu et al., 2016). Accordingly, stepwise BCCAO-induced CCH increased the cortical expression of proteins involved in protein synthesis and folding like glycine-tRNA ligase (GARS), heterogeneous nuclear ribonucleoprotein K (HNRNPK), nitrilase homolog 1 (NIT1), histidine triad nucleotide-binding protein 1 (HINT1), ATP-dependent RNA helicase DDX1, and the protein disulphide-isomerase A6 (PDIA6). Proteins involved in ubiquitin-mediated degradation also increased, including the COP9 signalosome complex subunit 2 (COPS2), the proteasome subunit alpha type-1 (PSMA1), the 26S protease regulatory subunit 6A (PSMC3), and the 26S protease regulatory subunit 6B (PSMC4) (Völgyi et al., 2017).

Genetic risk factors like the apolipoprotein E (APOE) gene cause vascular impairment (Farrer et al., 1997). In the elderly, mounting evidence suggests that AD links to atherosclerosis under brain hypoperfusion (de la Torre, 2002). In the general population, the e4 allele of the APOE gene poses the highest risk for sporadic AD (Farrer et al., 1997). It is a modest genetic risk factor for atherosclerosis (Wilson et al., 1996), associated with decreased efflux of cholesterol from cultured neurons (Michikawa et al., 2000). It might also boost APP-to-Aβ production (Casserly and Topol, 2004), contribute to proteostasis dysregulation impairing Aβ plaques' clearance, foster Aβ oligomer formation, and increase tau hyperphosphorylation (Casserly and Topol, 2004; Inbar et al., 2010; Argon and Gidalevitz, 2015).

## Neuroprotective agents targeting protein misfolding in CCH

Lipoxin A_4_ methyl ester (LXA_4_ ME) ameliorated hippocampal degeneration in rat under CCH induced by BCCAL and 2VO, attributable to regulation of ER stress and macroautophagy decreasing the level of C/EBP homologous protein, beclin-1, and the LC3-II-to-LC3-I ratio (Jia et al., 2015). Alternatively, the activation of the extracellular signal-regulated kinase/nuclear factor erythroid 2-related factor 2 (ERK/Nrf2) pathway might account for LXA_4_ ME neuroprotection (Jin et al., 2014). Also, baclofen has protective properties against hippocampal atrophy and neuronal apoptosis after CCH. Chronic treatment with baclofen induced suppression of the cytodestructive autophagic activity through protein kinase B (Akt)/ERK- B-cell lymphoma 2 (Bcl2)-beclin-1 signaling pathway and up-regulation of the protective autophagy activating the ionotropic metabotropic γ-aminobutyric acid (GABA)_A_ receptor-connexin (CX)_43_/CX_36_ signaling pathway. Since autophagy is a double-edged sword mechanism, bi-directional regulative effects on autophagy render neuroprotection (Liu et al., 2015). The modulation of autophagy also provides neuroprotection in a murine model of CCH through BCCAO. Treatment with the fatty acid amide hydrolase (FAAH) inhibitor URB597, might regulate autophagy, suppress apoptosis, and ameliorate ultrastructural neurodegeneration and cognitive decline in the CA1 area via the m-TOR pathway. This FAAH inhibitor also reversed the CCH-induced decrease in cannabinoid receptor (CB)1 level (Wang et al., 2017).

Melatonin administration modulated CCH-induced stress protein expression restoring chaperone HSP70 level in the hippocampus in a rat model of BCCAO (Ozacmak et al., 2009). N-stearoyl-L-tyrosine (NSTyr), an analog of the endogenous endocannabinoid anandamide (AEA), regulated the UPS and induced neuroprotection in rat hippocampus increasing proteasome peptidase activity and consequently inhibiting ubiquitinated proteins' intracellular aggregation (Hai et al., 2013). Previously, NSTyr had mitigated the cognitive deficits and restored hippocampal levels of the microtubule-associated protein 2 (MAP-2) and the synaptophysin protein in rats subjected to CCH (Lin et al., 2010). Besides, NSTyr induced neuroprotective effects on rat brain slices under OGD as well (Yao et al., 2009) (Table 1). In our laboratory, we have also found an AEA analog, Palmitoylethanolamide, could reverse behavioral dysfunctions and attenuate alterations in hippocampal MAP-2 levels in a murine model of acute hypoxia (Herrera et al., 2018). This experimental model of hypoxia is also known for inducing protein ubiquitination (Herrera et al., 2017).

**Table 1:** Neuroprotective agents targeting protein misfolding in CCH.

	**Agent**	**Pathway**	**Proteostatic complex**	**References**
NSTyr	N-stearoyl- L-tyrosine		UPS	Hai et al., 2013
LXA4 ME	Lipoxin A4 methyl ester	m-TOR	ER stress—macroautophagy	Jia et al., 2015
Melatonin		HSP70	HSP70	Ozacmak et al., 2009
Baclofen	GABA_B_ receptors agonist		Autophagy	Liu et al., 2015
URB597		m-TOR	Autophagy	Wang et al., 2017

*This table summarizes the most relevant findings on targeting protein misfolding in CCH over the last few years. Chronic Cerebral Hypoperfusion (CCH), Ubiquitine Proteosome System (UPS), Heat Shock Protein (HSP)*.

## Future directions

Neuroprotective targets in protein misfolding are represented by the different steps in the production and processing of proteins: synthesis, folding, repair, and degradation. The down-regulation of translation stands for an initial approach, aimed at reducing the load on molecular chaperones system. Enhancing and potentiating this system might represent an alternative approach since chaperones are responsible for the adequate protein folding and conformational repair when necessary. The up-regulation of the degrading pathways is another possibility along with non-toxic inclusions formation (Sweeney et al., 2017). Additionally, ER stress attenuation may protect from protein misfolding and aggregation. To date, increasing evidence pinpoints this strategy as a promising intervention in different animal models of neurodegeneration via genetic or pharmacological therapy (Hetz and Mollereau, 2014).

CCH is a chronic and silent disease characterized by years-standing of insufficient brain perfusion, concurrent with the worldwide highly prevalent MetS. Several studies were published using different models of CCH focusing mainly on inflammatory processes and cell death. Little research has paid attention to early signs of neurodegeneration like protein misfolding. The adequate function of the protein folding machinery critically depends on CBF and appears as a potential mechanism linking CCH with AD. With regard to CCH, active research is ongoing to uncover the mechanisms responsible for proteostatic network disbalance like the chaperones, UPR, and autophagy. Cellular autophagy is one of the most studied pathways of proteostatic cell response to insufficient brain blood supply. Future studies are encouraged to evaluate how mTOR affects autophagy and ER stress. Also, regulating the degree of eIF2α phosphorylation, which can be modified using specific compounds, may offer a promising approach to control ER stress in various diseases (Lindholm et al., 2017). Further investigation is required to explore the role of specific chaperones and the UPR system in CCH models. Conjointly, different animal models should be brought about to broaden our knowledge in this matters. Therapeutic drug options targeting misfolded proteins at different points in the proteostatic pathway are likely to emerge as promising neuroprotective treatments for cognitive impairment following CCH.

## Author contributions

MH: writing—original draft; LU: supervision; NT-U: supervision; CK: Supervision; JL: writing—review and editing; MO-L: original idea—review and editing—grammar, style and language; FC: funding acquisition, writing—review and editing.

### Conflict of interest statement

The authors declare that the research was conducted in the absence of any commercial or financial relationships that could be construed as a potential conflict of interest.
